# Genetic analysis of haptoglobin polymorphisms with cardiovascular disease and type 2 diabetes in the diabetes heart study

**DOI:** 10.1186/1475-2840-12-31

**Published:** 2013-02-11

**Authors:** Jeremy N Adams, Amanda J Cox, Barry I Freedman, Carl D Langefeld, J Jeffrey Carr, Donald W Bowden

**Affiliations:** 1 Molecular Genetics and Genomics Program, Wake Forest School of Medicine, Winston-Salem, North Carolina, 27157, USA; 2 Center for Genomics and Personalized Medicine Research, Wake Forest School of Medicine, Winston-Salem, North Carolina, 27157, USA; 3 Center for Diabetes Research, Wake Forest School of Medicine, Winston-Salem, North Carolina, 27157, USA; 4 Department of Biochemistry, Wake Forest School of Medicine, Medical Center Boulevard, Winston-Salem, North Carolina, 27157, USA; 5 Department of Internal Medicine – Nephrology, Wake Forest School of Medicine, Winston-Salem, North Carolina, 27157, USA; 6 Division of Public Health Sciences, Department of Biostatistical Sciences, Wake Forest School of Medicine, Winston-Salem, North Carolina, 27157, USA; 7 Department of Radiologic Sciences, Wake Forest School of Medicine, Winston-Salem, North Carolina, 27157, USA

**Keywords:** Haptoglobin, Genetic polymorphism, Cardiovascular disease, Type 2 diabetes

## Abstract

**Background:**

Haptoglobin (HP) is an acute phase protein that binds to freely circulating hemoglobin. HP exists as two distinct forms, HP1 and HP2. The longer HP2 form has been associated with cardiovascular (CVD) events and mortality in individuals with type 2 diabetes (T2DM).

**Methods:**

This study examined the association of *HP* genotypes with subclinical CVD, T2DM risk, and associated risk factors in a T2DM-enriched sample. Haptoglobin genotypes were determined in 1208 European Americans (EA) from 473 Diabetes Heart Study (DHS) families via PCR. Three promoter SNPs (rs5467, rs5470, and rs5471) were also genotyped.

**Results:**

Analyses revealed association between *HP2*-*2* duplication and increased carotid intima-media thickness (IMT; p = 0.001). No association between *HP* and measures of calcified arterial plaque were observed, but the *HP* polymorphism was associated with triglyceride concentrations (p = 0.005) and CVD mortality (p = 0.04). We found that the *HP2*-*2* genotype was associated with increased T2DM risk with an odds ratio (OR) of 1.49 (95% CI 1.18-1.86, p = 6.59x10^-4^). Promoter SNPs were not associated with any traits.

**Conclusions:**

This study suggests association between the *HP* duplication and IMT, triglycerides, CVD mortality, and T2DM in an EA population enriched for T2DM. Lack of association with atherosclerotic calcified plaque likely reflect differences in the pathogenesis of these CVD phenotypes. *HP* variation may contribute to the heritable risk for CVD complications in T2DM.

## Introduction

Cardiovascular disease (CVD) is one of the major complications associated with type 2 diabetes mellitus (T2DM). As of 2011, 25.8 million Americans had diagnosed T2DM [1]. More than 50% of individuals with T2DM had coronary heart disease, stroke, or cardiac disease [2]. T2DM is an independent risk factor for development of CVD with the relative risk of CVD mortality of 2.1 in men and 4.9 in women, relative to non-T2DM affected individuals [3,4]. There is increasing evidence that genetic and environmental factors contribute to this risk.

Haptoglobin (HP) is a 54 kDa protein, found abundantly in the serum [5,6]. The *HP* gene has two major alleles: *HP1*, (containing five exons) and *HP2*, (containing seven exons) which likely arose from a duplication event involving exons 3 and 4, producing a 61 kDa protein [6]. In its ancestral form, HP is a dimer, however, the *HP 12* encoded protein exists as linear polymers containing 2–8 monomers, while the *HP 22* encoded protein exists as circular polymers of 3–10 Hp monomers [6]. The expanded polymerization in the *HP 12* and *HP 22* genotypes is due to the duplication of the multimerization domain in exon 3 [6]. Genotype frequencies vary in different ethnicities. In European Americans (EA) they have been reported as 16% *HP 11*, 48% *HP 12*, and 36% *HP 22*[5].

HP may prevent oxidative damage through mechanisms including stabilization of the heme iron within hemoglobin (Hb) [7]. The HP-Hb complex is rapidly removed from circulation via CD163 mediated endocytosis by hepatic Kupfer cells [8]. The HP 1–1 protein is both more efficient than HP 2–2 at preventing oxidation caused by the heme iron [9] and is internalized and cleared from circulation more rapidly; with half lives of approximately 20 minutes and 50 minutes for HP 1-1-Hb and HP 2-2-Hb respectively [10,11]. Binding of the HP-Hb complex to CD163 induces the production of several cytokines and anti-inflammatory mediators [9,12] with a much larger production of anti-inflammatory mediators induced by HP 1–1 compared to HP 2–2 [13,14].

HP has been implicated in both T2DM and T2DM-associated CVD [15,16]. In the latter context the binding of HP to apolipoprotein A1 (ApoA1) has also been reported [17]. HP binds to ApoA1 in the same location as lecithin-cholesterol acyltransferase (LCAT), subsequently decreasing LCAT activity and therefore limiting high density lipoprotein (HDL) maturation. This inhibits reverse cholesterol transport causing HDL to become proatherogenic [17]. In addition, the tethering of Hb to HDL via the HP-ApoA1 allows the oxidation of HDL and its acquisition of proatherogenic and proinflammatory properties [18]. Due to the multimerization of the Hp 2 protein, individuals with the *HP* 2–2 genotype have significantly more HP attached to HDL via ApoA1 increasing these properties [11].

Due to the striking differences in properties of the HP 1 and HP 2 proteins, several studies have investigated the impact of the HP phenotype on CVD risk. There have been differing results when examining different populations and different outcomes. Studies investigating incident CVD in individuals affected by T2DM show an increased risk with the HP 2–2. One study [19] found that individuals with T2DM and the *HP22* genotype had increased risk for CVD events. In addition, Suleiman *et al*. in 2005 [20] found that individuals with T2DM and the HP 1–1 phenotype had decreased 30-day mortality and heart failure after acute myocardial infarction compared to individuals with the HP 2–2 phenotype, again suggesting the HP 2-2 phenotype as the risk phenotype. This association was not seen in individuals without T2DM. Indeed similar observations have been made in type 1 diabetes; Simpson *et al*. [21] found that in individuals with type 1 diabetes the *HP 22* genotype predicted coronary artery calcification progression, a measure of subclinical CVD. In contrast, in cohorts where rates of T2DM are low or individuals with T2DM excluded, the *HP11* genotype has been shown to be associated with an increased risk for mortality due to coronary heart disease [22]. Similarly, in the Framingham offspring study the HP 1–2 or HP 2–2 phenotypes were associated with decreased rates of prevalent CHD [23].

The *HP* duplication has also been examined for association with T2DM. The role of HP in regulation of inflammation suggests a potential role in T2DM pathogenesis. There are several studies showing that the *HP* duplication was associated with T2DM risk in different populations [24,25].

Thus, the relationship between *HP* polymorphism and CVD in T2DM-affected individuals is likely complex and association with T2D risk has been documented to a limited degree. Based on these prior studies we hypothesized that if the *HP 22* genotype is associated with CVD events in people with T2DM, then a similar association would likely be observed with measures of subclinical CVD in predominately T2DM-affected populations. We have taken advantage of the richly phenotyped Diabetes Heart Study [26] (DHS) sample with measures of coronary artery calcification (CAC; or calcified plaque), carotid wall intima-medial thickness (IMT), and blood lipid traits to investigate this hypothesis. Further, the DHS provides a base from which to investigate whether the *HP* locus is directly associated with T2DM risk.

## Methods

### Subjects

The DHS is a study of the genetic and epidemiological causes of CVD in individuals with T2DM. Ascertainment, recruitment, and examination have been previously described in detail [26]. Briefly, siblings concordant for T2DM and without serious health conditions, *e*.*g*. renal replacement therapy, were recruited. T2DM was defined as diabetes developing after 35 years of age, treatment with insulin and/or oral agents and absence of historical evidence of ketoacidosis. If available, additional non-T2DM affected siblings were recruited simultaneously using criteria above to exclude T2DM. The 1208 DHS EA individuals used in this analysis were from 473 families.

### Clinical evaluation

The protocols for this study were approved by the Institutional Review Board at Wake Forest School of Medicine; written informed consent was received prior to participation. Examinations were conducted in the General Clinical Research Center of the Wake Forest Baptist Medical Center, and included interviews for medical history and health behaviors, anthropometric measures, resting blood pressure, electrocardiography, fasting blood sampling and spot urine collection. Individuals reported history of prior CVD based on prior event (angina, myocardial infarction, stroke) and/or intervention (coronary angiography, coronary artery bypass grafting, carotid endarterectomy). CAC, carotid artery calcified plaque (CarCP) and infra-renal abdominal aortic calcified plaque (AACP) were measured using fast-gated helical CT scanning, and calcium scores calculated as previously described and reported as an Agatston score [27,28]. Carotid IMT was measured by high-resolution B-mode ultrasonography with a 7.5-MHz transducer and a Biosound Esaote (AU5) ultrasound machine (Biosound Esaote, Inc., Indianapolis, IN) as previously described [29]. All measurements were not available for all participants.

### Mortality

Vital status was determined from the National Social Security Death Index maintained by the United States Social Security Administration. For participants confirmed as deceased, length of follow-up was determined from data of the initial study visit to date of death. For deceased participants, copies of death certificates were obtained from relevant county Vital Records Offices to confirm cause of death. For all other participants the length of follow-up was determined from the date of the initial study visit to January 1, 2011. Cause of death was categorized based on information contained in death certificates as CVD-related (myocardial infarction, congestive heart failure, cardiac arrhythmia, sudden cardiac death, peripheral vascular disease, and stroke) or cancer, infection, end-stage renal disease, accidental, or other (including obstructive pulmonary disease, pulmonary fibrosis, liver failure and Alzheimer’s dementia).

### Genotyping

Genomic DNA was purified from whole-blood samples obtained from subjects using the PUREGENE DNA isolation kit (Gentra Systems., Minneapolis, MN). DNA was quantitated using standardized fluorometric readings on a Hoefer DyNA Quant 200 fluorometer (Hoefer Pharmacia Biotech, Inc., San Francisco, CA). Each sample was diluted to a final concentration of 20 ng/μL. *HP* duplication genotyping was performed using paired polymerase chain reactions (PCR). PCR primers, reaction and cycling conditions were performed as described previously by Koch *et al*. [30] for 10 μL reactions containing 45–60 ng DNA and primers at final concentrations of 0.8 μM for the *HP1* and 0.6 μM for the *HP2*.

**Figure 1:**
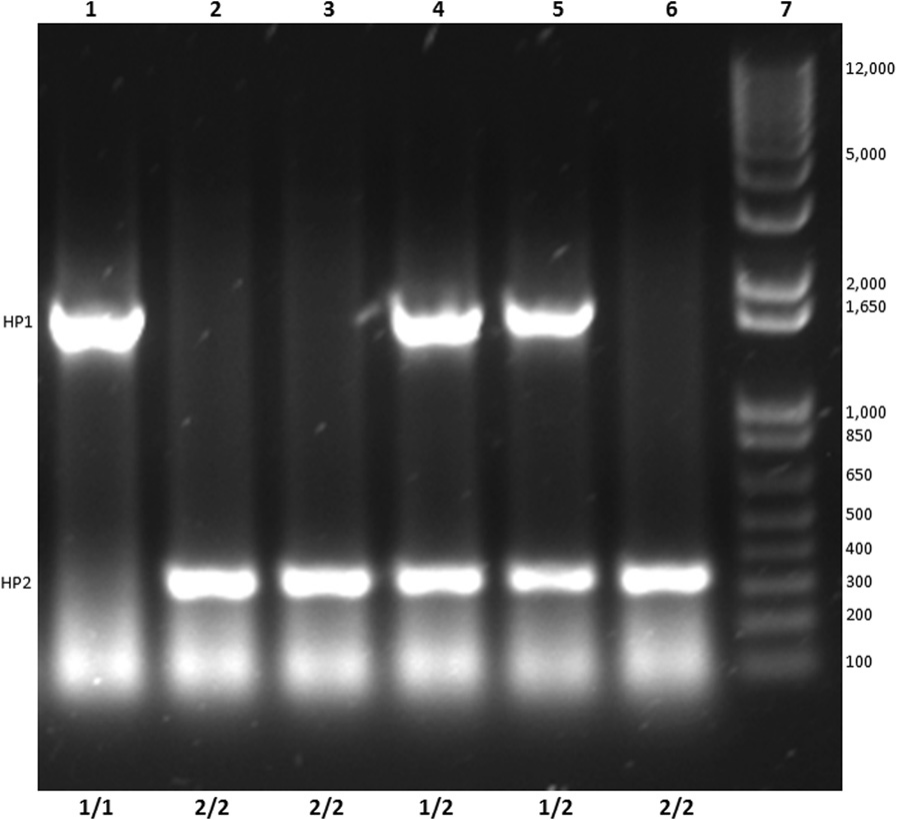
**Gel picture of *HP *PCR products.** Example of *HP* genotype discrimination using PCR reaction products resolved on a 1.4% agarose gel with ethidium bromide staining where lanes 1–6 are participant samples and lane 7 a DNA MW size ladder. Lane numbers are at the top and genotyping call is at the bottom.

PCR products from the two reactions were combined (10 μL of the *HP1* product with 5 μL of the *HP2* PCR product) and resolved on a 1% agarose gel (Figure 1) which was visualized by staining with 1.4% ethidium bromide and the images captured using an Alpha Imager (Alpha Innotech, San Leandro, CA). Haptoglobin genotypes were called independently by two investigators (JNA and AJC) with 100% concordance between calls. Genotyping also included a total of 29 blind duplicates to allow for evaluation of genotyping accuracy. The concordance rate for these blind duplicates was 100%.

### *HP* promoter polymorphism genotyping

In addition to the classical *HP1* and *HP2* variants, three single nucleotide polymorphisms (SNPs), rs5467, rs5470, and rs5471, located in the promoter region of *HP* and previously reported to be associated with circulating HP, were genotyped in the DHS. Genotyping was performed using the Sequenom Mass ARRAY genotyping system (Sequenom, San Diego, CA) and PCR primers were designed using the Mass ARRAY Assay Design 3.4 Software (Sequenom). These SNPs are in low linkage disequilibrium (LD) with each other or with the *HP* duplication (r^2^ ≤0.03) (see Additional file 1). An additional 41 quality control samples were included in the genotyping analysis to serve as blind duplicates. The concordance rate for these blind duplicates was 100%. The minimum acceptable call frequency for all SNPs was 95%. The average call frequency was 98.4 ± 0.001% (mean ± SD). Samples with genotyping efficiency rates <90% were excluded from further analysis.

### T2DM-affected cases and controls for analysis of T2DM risk

An additional 606 EA T2DM cases and 985 EA non-T2DM affected controls from an independent study of EA T2DM [31,32] were genotyped for the *HP* duplication using the PCR method described above. Thirty-one additional quality control samples were included as blind duplicates. The concordance rate for these samples was 100%. For analysis we combined *HP* data for unrelated T2DM cases from DHS (n = 473) with T2DM cases from the independent T2DM case sample for a total of 1079 cases. These data were evaluated for association with *HP* genotypes contrasting with controls without T2DM (n = 985).

### Statistical analysis

*HP* allele frequencies were calculated for a sub-set of unrelated individuals and departure from Hardy-Weinberg equilibrium was calculated from a group of unrelated samples using a chi-squared goodness-of-fit test. Association between the *HP* genotypes and subclinical CVD measures was examined using variance component methods computed using SOLAR v4.3.1 (Texas Biomedical Research Institute, San Antonio, TX, USA). Each trait was examined using additive, dominant, and recessive models of inheritance. Continuous variables were transformed prior to analysis to approximate conditional normality. Age, gender, and T2DM-affection status were used as covariates in analysis of quantitative traits. Additional covariates (smoking, hypertension, C-reactive protein, lipid medication use, hypertension medication use, and T2DM duration) were also evaluated, but did not impact the results. Analyses were repeated in the subset of the population that was affected by T2DM. Statistical significance was accepted at p < 0.05. The program SNPGWA (http://www.phs.wfubmc.edu/public_bios/sec_gene/downloads.cfm) [33] was used to test for associations between *HP* and T2DM risk comparing individuals affected with T2DM (DHS T2DM-affected unrelated individuals [n = 473] and EA T2DM-affected individuals [n = 606]) to the EA non-T2DM affected controls (n = 985). Covariates used in this analysis were age, gender, and body mass index (BMI). Analyses included samples that had genotype and all covariate data.

## Results

Characteristics of the DHS sample are summarized in Table 1 including the mean trait values for the overall sample and for each *HP* genotype group. Briefly, the mean age of the sample was 61.5 years; 1013 individuals (83.86%) were T2DM-affected and slightly more than 50% (643) were female. Overall, the characteristics of the DHS sample are representative of T2DM-affected patients in the general population.

**Table 1:** **Demographic characteristics of DHS samples, including *HP *duplication genotypes (data shown are mean ± SD, unless specified otherwise)**

**DHS Demographics**				
		***HP *Genotypes**		
	**Total**	**1/1**	**1/2**	**2/2**
Number	1208	168	505	535
Age (years)	61.5 ± 9.35	62.5 ± 9.36	61.2 ± 9.34	61.5 ± 9.36
Female (%)	643 (53.2)	87 (51.8)	274 (54.3)	282 (52.7)
BMI (kg/m^2^)	31.8 ± 6.49	31.9 ± 6.27	32.1 ± 6.81	31.4 ± 6.23
Affected (%)	1013 (83.9)	134 (79.8)	431 (85.4)	448 (83.7)
Diabetes duration (years)	10.41 ± 7.1	10.95 ± 7.9	10.15 ± 7.24	10.49 ± 6.86
Metabolic Syndrome (%)	1029 (85.2)	140 (83.3)	434 (85.9)	455 (85.1)
Glucose (mg/dL)	139.4 ± 55.5	139.7 ± 58.8	142.3 ± 56.7	136.7 ± 53.1
HbA1c (%)	7.29 ± 1.7	7.13 ± 1.57	7.31 ± 1.78	7.32 ± 1.78
C-reactive protein (mg/dl)	0.59 ± 0.97	0.54 ± 0.81	0.60 ± 1.05	0.59 ± 0.94
Hypertension (%)	1025 (84.85)	141 (83.93)	422 (83.56)	462 (86.36)
Smoking (%)	708 (58.85)	96 (57.49)	285 (56.55)	327 (61.47)
Lipid medication (%)	540 (44.7)	76 (45.24)	221 (43.76)	243 (45.42)
Anti-hypertensive medication (%)	747 (61.84)	101 (60.12)	324 (64.16)	322 (60.19)
CAC	1662.5 ± 3160.7	1679.1 ± 2449.8	1607.2 ± 3001.5	1708.5 ± 3492.3
CarCP	312.9 ± 672.1	276.2 ± 522.5	292.8 ± 610.8	343.3 ± 762.4
AACP	10949.3 ± 15748.8	12628.86 ± 16231.1	9781.9 ± 14236.0	11458.1 ± 16775.3
IMT (mm)	0.676 ± 0.134	0.677 ± 0.136	0.665 ± 0.129	0.686 ± 0.139
Cholesterol (mg/dL)	186.8 ± 42.4	187.5 ± 46.2	187.7 ± 43.4	185.8 ± 40.3
LDL (mg/dL)	105.1 ± 32.7	104.0 ± 35.3	103.3 ± 30.8	107.1 ± 33.5
HDL (mg/dL)	43.1 ± 12.5	43.5 ± 14.4	43.3 ± 12.7	42.7 ± 11.6
Triglycerides (mg/dL)	201.4 ± 132.1	212.8 ± 162.3	208.8 ± 133.7	190.9 ± 119.2
History of CVD (%)	471 (38.99)	72 (42.86)	191 (37.82)	208 (38.88)
Non-CVD Mortality (%)	122 (10.10)	19 (11.31)	49 (9.70)	54 (10.09)
CVD Mortality (%)	100 (8.28)	11 (6.55)	34 (6.73)	55 (10.28)

*HP* duplication genotypes were determined for 1208 individuals (Table 1). The genotype frequencies were 13.9% *HP 1*–*1*, 41.8% *HP 1*–*2*, and 44.3% *HP 2*–2 and were consistent with Hardy-Weinberg proportions.

### CVD associations

*HP* duplication data was analyzed for association with multiple CVD measures, risk factors, and outcomes: calcified plaque (CAC, CarCP, and AACP), carotid IMT, lipids, prevalent CVD and mortality. The *HP* duplication was associated with carotid IMT (p = 0.001, Table 2). We did not, however, observe a significant association of *HP* with measures of vascular calcification: CAC, CarCP or AACP (Table 2). There did not appear to be any discernable trend of increased calcification in the mean trait values by genotype for any of the three arterial beds (Table 1). Additional analyses performed in T2DM-affected individuals alone, revealed similar results (data not shown).

**Table 2:** **Association result for the *HP *duplication with measures of subclinical CVD and blood lipids**

***HP *Duplication – CVD Association Results**							
		**Additive**		**Dominant**		**Recessive**	
	**N**	**Beta ± SE**	**p-value**	**Beta ± SE**	**p-value**	**Beta ± SE**	**p-value**
CAC	1134	0.022±0.097	0.82	−0.025±0.135	0.85	0.135±0.189	0.48
CarCP	1142	−0.159±0.102	0.12	−0.180±0.142	0.21	−0.255±0.200	0.20
AACP	865	−0.025±0.102	0.81	−0.117±0.141	0.41	0.141±0.202	0.48
IMT	1103	−0.008±0.003	**0.01**	−0.014±0.004	**0.001**	−0.002±0.006	0.79
Cholesterol	1188	0.006±0.010	0.53	0.014±0.013	0.28	−0.005±0.019	0.78
Triglycerides	1188	0.060±0.024	**0.01**	0.091±0.033	**0.005**	0.050±0.047	0.28
Prevalent CVD	1084	−0.033 ± 0.061	0.58	0.006 ± 0.083	0.94	−0.140 ± 0.117	0.23
All Cause Mortality	1208	0.063 ± 0.065	0.33	0.123 ± 0.089	0.17	−0.004 ± 0.129	0.98
CVD Mortality	1208	0.145 ± 0.081	0.07	0.222 ± 0.101	**0.04**	0.117 ± 0.116	0.47

Associations were examined under additive, dominant and recessive genetic models. Bold indicates statistical significance. CAC: coronary artery calcified plaque; CarCP: carotid artery calcified plaque; AACP: abdominal aortic calcified plaque; IMT: carotid intima-media thickness. SE = standard error.

A total of 471 (39.0%) individuals in the DHS have a self reported history of prior CVD. This included 72 individuals (42.9%) for *HP 1*–*1*, for *HP 1*–*2* genotype 191 (37.8%), and for *HP 2*–*2* 208 (38.98%). However, prevalent CVD was not associated with the *HP* duplication (p = 0.23-0.94; Table 2).

The *HP2* allele was associated with serum triglyceride concentrations (p = 0.005; Table 2). With each additional copy of the *HP2* allele, triglyceride concentrations decreased and were on average 10.3 mg/dL lower with the *HP2*-*2* genotype (Table 1). In addition, the *HP2* allele was not associated with all cause mortality (p = 0.17); however, it was nominally associated with CVD mortality (p = 0.04; Table 2). Results were essentially unchanged when analyses were repeated in T2DM affected individuals only (see Additional file 2).

### *HP* promoter polymorphisms

SNPs rs5467, rs5470, and rs5471 were genotyped based on prior reports that they were associated with HP protein concentrations [34,35]. Each SNP was in Hardy-Weinberg equilibrium. Minor allele frequencies (MAF) were 19.2% for rs5467, 0.04% for rs5470, and 0.08% for rs5471. None of these *HP* SNPs were associated with any of the subclinical CVD traits, CVD risk factors, events, or mortality ascertained in the current study (see Additional file 3).

### *HP* polymorphism and T2DM risk

Table 3 contains the clinical demographics of the T2DM case and control samples. This sample was ascertained and recruited independently from the DHS, but using the same diagnostic criteria for T2DM diagnosis and has been the basis for prior genetic studies of T2DM [31,32]. Briefly, the mean age of the T2DM-affected case sample was 65.2 ± 9.9 years compared to 53.8 ±15.0 for the non-T2DM controls. Fewer than 50% (n = 299) of cases and greater than 63% (n = 628) of the controls were female.

**Table 3:** **Demographic characteristics of T2DM case and non-diabetic control samples, including *HP *duplication genotype measures (data shown is mean ± SD unless specified otherwise)**

**T2DM Demographics**					
	**T2DM Cases**	**DHS T2DM Cases**	**Total T2DM Cases**	**Non-diabetes controls**	**Total**
Number	606	473	1079	985	2064
Age (years)	65.18 ± 10.45	62.09 ± 8.86	63.79 ± 9.88	53.83 ± 15.03	59.82 ± 13.13
Female (%)	299 (49.42)	246 (52.01)	545 (50.06)	628 (63.82)	1173 (56.89)
BMI (kg/m2)	29.65 ± 7.15	32.64 ± 6.53	30.97 ± 7.04	28.35 ± 5.67	29.97 ± 6.67
Affected (%)	606 (100)	473 (100)	1079 (100)	0 (0.00)	1079 (52.13)
1/1 Genotype (%)	88 (14.52)	64 (13.53)	152 (14.09)	156 (15.84)	308 (14.92)
1/2 Genotype (%)	274 (45.21)	194 (41.01)	468 (43.37)	493 (50.05)	961 (46.66)
2/2 Genotype (%)	244 (40.26)	215 (45.45)	459 (42.53)	336 (34.11)	795 (38.52)

For all T2DM cases, genotype frequencies were 14.1% *HP 1*–*1*, 43.4% *HP 1*–*2*, and 42.5% *HP 2*–*2*. In non-T2DM controls, genotype frequencies were 15.8% *HP 1*–*1*, 50.1% *HP 1*–*2*, and 34.1% *HP 2*–*2* (Table 3). Genotype frequencies in both samples were consistent with Hardy-Weinberg proportions. Individuals with the *HP 2*–*2* genotype were found to be more likely to have T2DM (recessive model OR: 1.49; 95% CI: 1.18-1.86; p = 6.59x10^-4^). Similar to the DHS analysis of CVD, there was no evidence of association of T2DM risk with *HP* promoter SNPs in the DHS (data not shown).

## Discussion

This study evaluated association of *HP* gene polymorphisms with subclinical CVD, mortality, and T2DM in 1208 EA individuals from the DHS. The *HP 2*–*2* genotype was associated with increased carotid IMT (p = 0.001, Table 1) in this T2DM enriched population. However, we did not observe significant evidence of association between *HP* genotype and calcified plaque as a different measure of subclinical CVD. An association with triglyceride concentrations was also observed; the *HP 2*–*2* genotype was associated with lower concentrations. The biological mechanism for this latter association is as of yet, unknown. We also observed suggestive evidence for association of the *HP* duplication polymorphism with CVD related mortality in the DHS. In addition, we found that the *HP 2*–*2* genotype was associated with T2DM status (OR: 1.49; 95% CI: 1.18-1.86; p = 6.59x10^-4^).

Several prior studies have investigated *HP* polymorphisms and CVD risk in T2DM. In 2002 Levy *et al*. [19] reported an OR of CVD events in diabetes five times greater with the HP 2–2 phenotype, than with HP 1–1 in a study that included 206 CVD patients and 206 CVD controls (146 and 93 were affected by T2DM, respectively, as part of the Strong Heart Study). In 2004, a subsequent study by Levy *et al*. [23] included 3273 individuals in the Framingham Heart Study, however only a subset of 433 individuals were affected with T2DM, and of these, only 86 had a history of prevalent CVD. Finally, a 2003 study in individuals with acute myocardial infarction (AMI) reported individuals with T2DM and the *HP2* allele had increased mortality following AMI compared to individuals with T2DM and the *HP 11* genotype (included only 224 T2DM-affected individuals) [20]. In the present study we detected modest evidence of association with carotid IMT, but did not strongly replicate association with history of prior CVD and only nominally with CVD mortality. Parenthetically, IMT and measures of vascular calcification are not highly correlated [26]. The DHS is predominately comprised of T2DM-affected subjects (1013 of 1208 participants). Our primary measures were the subclinical measures of CVD, CAC and IMT which may not be as strongly influenced by *HP* polymorphism. Of the DHS subjects, 435 were T2DM-affected participants with a history of prevalent CVD, based upon self-reported history and prior intervention which was not associated with *HP* genotype. The analysis with CVD mortality, a firm endpoint, suggests a possible contribution to risk. Given the association of the *HP 22* genotype with risk for mortality, it is possible that a survival bias may be present. However, genotype frequencies were consistent with Hardy-Weinberg equilibrium. In addition, the genotype frequencies of the *HP* duplication in this study were similar to those reported previously [5].

In prior reports, two promoter SNPs, rs5470 and rs5471 were associated with altered levels of *HP* expression [34,35] with rs5471 reported to be associated with the Haptoglobin 1–2 modified (HP1-2mod) phenotype. In individuals with the rs5471 “C” allele and the *HP 12* genotype, normal expression levels of the HP 1 protein, but decreased levels of HP 2 have been reported [34]. It has been suggested that the decreased levels of HP 2 lead to greater oxidative stress [36]. We did not observe evidence of association for these three genotyped HP SNPs genotyped (rs5467, rs5470, and rs5471) with measures of subclinical CVD, history of CVD, or mortality. One possible explanation is the low MAF for both rs5470, and rs5471 (0.0004 and 0.0008 respectively). The combination of the *HP12* and the rs5471 SNP has been reported in approximately 10% of African Americans [34], but we are unaware of reports of the frequency of the HP1-2mod phenotype in other populations. In this study there were no minor allele homozygotes for rs5471, nor rs5471 heterozygotes with the *HP12* genotype; as such, the Haptoglobin 1–2 modified phenotype is unlikely to have confounded the *HP* associations described here. In addition, in LD analysis (see Additional file 1) these two SNPs were in low LD with the *HP* duplication. Thus these two promoter SNPs along with rs5467 probably do not have an impact on CVD or T2DM status. However, there are other SNPs that are known to impact circulating HP concentrations (e.g. rs2000999) [37] that we did not genotype in the current study which may also contribute to the variance in HP and its role in CVD risk. The lack of measured HP concentrations in the DHS and the inability to further control for these additional genetic variants is one limitation of this work.

Several previous studies have investigated the effect of the HP polymorphism on T2DM risk. A previous study by Stern *et al*. in 1986 [24] found that the *HP1* allele was associated diabetes risk in Mexican Americans. They found that a single copy of the *HP1* allele increased T2DM risk by 50% and a second copy increased risk by 100%. A second report from 2006 by Quaye *et al*. [25] found that the HP 2–2 phenotype was a risk factor for T2DM in a population in Ghana. The current study had a larger sample size than either of the two previous studies, albeit in a different ethnicity. In this study it was found that EA individuals with the *HP 2*–2 genotype are more likely to have T2DM with an OR of 1.49. These studies, when combined suggest that *HP* is a risk gene for diabetes or is in LD with a risk gene. Several studies have shown that the different alleles lead to different levels of circulating HP protein [22,37]. Higher circulating HP has been suggested to be associated with metabolic syndrome, high blood pressure, and elevated glucose [38]. This could possibly explain the association of the HP polymorphism with T2DM. Different risk alleles across populations are problematic as it could be difficult to assess risk across different populations.

Importantly, the T2DM association may be difficult to further investigate without subsequent data generation since the duplication is not included in the major, publically available, databases. Furthermore, any subsequent studies will require a targeted phenotyping approach either through analysis of HP in serum or genotyping through fragment size analysis as performed in this study, since the duplication is not captured by the current commercially available genome-wide genotyping platforms and does not appear to be tagged by other common polymorphisms [37]. A genome wide association study (GWAS) analysis has been performed in the DHS and LD was analyzed for the *HP* duplication and the two SNPs on the GWAS chip that are closest to the duplication (rs16973636 and rs2287998). The SNPs were found to have low LD with the duplication with an r^2^ of ≤0.01 (data not shown).

## Conclusions

Overall, we detected limited association of haptoglobin polymorphisms with CVD. The *HP22* genotype was found to be positively associated with carotid IMT and the *HP2* allele was associated with decreased serum triglyceride concentrations. We identified an association with T2DM in EAs that has not been reported previously [39-41]. Further studies are needed to extend and replicate these relationships.

## Abbreviations

AACP: Infra-renal abdominal aortic calcified plaque; ApoA1: Apolipoprotein A1; BMI: Body mass index; CAC: Coronary artery calcified plaque; CarCP: Carotid artery calcified plaque; CVD: Cardiovascular disease; DHS: Diabetes Heart Study; EA: European American; GWAS: Genome wide association study; Hb: Hemoglobin; HDL: High density lipoprotein; HP: Haptoglobin; IMT: Intima media thickness; LCAT: Lecithin-cholesterol acyltransferase; LD: Linkage disequilibrium; MAF: Minor allele frequency; OR: Odds ratio; PCR: Polymerase chain reaction; SNP: Single nucleotide polymorphism; T2DM: Type 2 diabetes mellitus.

## Competing interests

The authors declare they have no competing interests.

## Authors’ contributions

JNA perfomed the duplication genotyping and statistical analysis and wrote the manuscript; AJC performed the SNP genotyping, assisted with the duplication genotyping, and assisted with the manuscript preparation; BIF was involved in the conception of the DHS, participated in subject recruitment and clinical assessment and reviewed the manuscript; CLD contributed to statistical analyses and reviewed the manuscript; JJC was involved in the conception of the DHS, participated in subject recruitment and clinical assessment, and reviewed the manuscript; DWB designed and supervised the DHS, conceived the haptoglobin investigation and assisted with the manuscript preparation. All authors read and approved the final manuscript.

## Supplementary Material

Additional file 1 **LD of *HP*duplication and promoter SNPs.** LD plot showing r^2^ between the *HP* duplication (HPDup) and genotyped promoter SNPs (rs5467, rs5470, and rs5471) based on genotypes from the DHS sample. Click here for file

Additional file 2 ***HP*Duplication – T2DM CVD Association Results.** Association results for the *HP* duplication in the individuals with T2DM only with measures of subclinical CVD (CAC: coronary artery calcified plaque; CarCP: carotid artery calcified plaque; AACP: abdominal aortic calcified plaque; IMT: carotid intima-media thickness) and blood lipids. Associations were examined under additive, dominant and recessive genetic models. Bold indicates statistical significance. SE = standard error. Click here for file

Additional file 3 ***HP*Promoter SNP – CVD Association Results.** Association analysis of *HP* promoter SNPs with subclinical CVD (CAC: coronary artery calcified plaque; CarCP: carotid artery calcified plaque; AACP: abdominal aortic calcified plaque; IMT: carotid intima-media thickness) and blood lipids. Associations were examined under an additive model. SE = standard error. Click here for file
