# Supplementary figures and images for: Genetic analysis of haptoglobin polymorphisms with cardiovascular disease and type 2 diabetes in the diabetes heart study
Source: Cardiovasc Diabetol. 2013 Feb 11;12:31. doi: 10.1186/1475-2840-12-31 (PMC3576297; doi:10.1186/1475-2840-12-31)

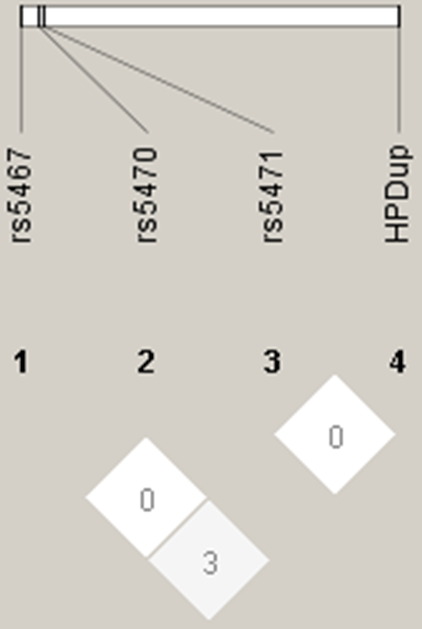

Supplement: Additional file 1 — LD of HPduplication and promoter SNPs. LD plot showing r2 between the HP duplication (HPDup) and genotyped promoter SNPs (rs5467, rs5470, and rs5471) based on genotypes from the DHS sample. [file 1475-2840-12-31-S1.png]
